# Predictors of time to claim closure following a non-catastrophic injury sustained in a motor vehicle crash: a prospective cohort study

**DOI:** 10.1186/s12889-016-3093-y

**Published:** 2016-05-20

**Authors:** Bamini Gopinath, Nieke A. Elbers, Jagnoor Jagnoor, Ian A. Harris, Michael Nicholas, Petrina Casey, Fiona Blyth, Christopher G. Maher, Ian D. Cameron

**Affiliations:** John Walsh Centre for Rehabilitation Studies, Kolling Institute of Medical Research, University of Sydney, Corner Reserve Road & First Avenue, Royal North Shore Hospital, St Leonards, NSW, 2065 Australia; Ingham Institute for Applied Medical Research, University of New South Wales, New South Wales, Australia; Pain Management Research Institute, Sydney Medical School, University of Sydney, Sydney, Australia; School of Public Health, University of Sydney, Sydney, Australia; George Institute for Global Health, Sydney Medical School, University of Sydney, Sydney, Australia

**Keywords:** Minor injury, Road traffic crash, Claim closure, Compensation, Cohort, Predictors

## Abstract

**Background:**

Research suggests that exposure to the compensation system (including time to case closure) could adversely influence a persons’ recovery following injury. However, the long-term predictors of time to claim closure following minor road traffic injuries remain unclear. We aimed to assess a wide spectrum of factors that could influence time to claim closure (socio-demographic, compensation-related, health, psychosocial and pre-injury factors) over 24 months following a non-catastrophic injury.

**Methods:**

Prospective cohort study of 364 participants involved in a compensation scheme following a motor vehicle crash. We used a telephone-administered questionnaire to obtain information on potential explanatory variables. Information on time to claim closure was obtained from an insurance regulatory authority maintained database, and was classified as the duration between the crash date and claim settlement date, and categorized into < 12 (early), > 12–24 (medium) and > 24 months (late).

**Results:**

Just over half of claimants (54 %) had settled their claim by 12 months, while 17 % and 30 % took > 12–24 months and > 24 months for claim closure, respectively. Whiplash at baseline was associated with claim closure time of > 12–24 months versus < 12 months: multivariable-adjusted OR 2.38 (95 % CI 1.06–5.39). Claimants who were overweight/obese versus normal/underweight at the time of injury were ~3.0-fold more likely to settle their claim at > 12–24 months than < 12 months. Consulting a lawyer was associated with a 10.4- and 21.0-fold increased likelihood of settling a claim at > 12–24 months and > 24 months, respectively. Each 1-unit increase in Orebro Musculoskeletal Pain Screening Questionnaire scores at baseline was associated with greater odds of both medium (> 12–24 months) and delayed claim settlement date (> 24 months): multivariable-adjusted OR 1.04 (95 % CU 1.01–1.07) and 1.02 (95 % CI 1.00–1.05), respectively.

**Conclusions:**

Around a third of claimants with a minor injury had not settled by 24 months. Health-related factors and lawyer involvement independently influenced time to claim closure.

## Background

There is increasing evidence to show that claiming compensation and prolonged exposure to the compensation system are prognostic indicators of poorer recovery and negative health outcomes among those who have sustained an injury in a motor vehicle crash [1–8]. However, the reasons underlying the associations between the compensation process and poorer health and delayed recovery remain unclear. Some researchers have speculated that people who claim compensation compared to those who do not claim could have different personal traits or characteristics such as a worse pre-injury health status and lower socio-economic status profile, and that these characteristics could contribute to poorer recovery in the longer term [8, 9]. Others suggest the contribution of secondary gain or accident neurosis, positing that claimants do not recover because of a financial incentive not to get better as long as the process lasts [10]. Finally, prolonged exposure to the scheme increases the likelihood of participants coming into contact with system complexities which are known to be stressful [9] including; numerous assessments [11] and the overall adversarial nature of contacts with claims staff [12, 13]. However, the direction of the relationship between claiming compensation and negative outcomes remains unclear [14], that is, does exposure (and length of exposure) to the compensation system cause poor health or does a poor health profile (or other factors) lead to prolonged exposure to the compensation system [12]?

While an extensive amount of research has been conducted regarding the predictors of health outcomes and recovery following a minor injury sustained in a motor vehicle crash, there is less published literature on the independent predictors of time to claim closure. In a Canadian cohort of whiplash claimants, pain severity, physical functioning, and depressive symptomology were strongly associated with prolonged time-to-claim closure [15]. Recently, *Casey* et al. [12] showed that higher initial disability, prior compensation claim, poorer mental wellbeing, and legal involvement were factors that delayed claim closure in an Australian cohort of people with whiplash associated disorder.

We aimed to explore the prospective association between socio-demographic, compensation, psychological, pre-injury, health- and injury-related characteristics that were independently associated with time to claim closure among claimants who sustained non-catastrophic injuries in a motor vehicle crash. These study findings are potentially useful as they could contribute to building a stronger case for insurers or claims handlers to include bio-psychosocial health outcome information as part of their routine data collection systems, thereby, increasing knowledge on the longer term health profile of their injured clients [12].

## Methods

### Study population

Potential participants were identified from the New South Wales (NSW) State Insurance Regulatory Authority (SIRA) Personal Injury Registry (previously known as Motor Accidents Authority) database. The injury database consists of people who made claims on the Compulsory Third Party scheme through the Accident Notification Form or from the Personal Injury Claim Form. The Accident Notification Form is for a limited insurance claim that provides early payment of reasonable and necessary medical expenses, and/or lost earnings up, to a maximum of AU$5,000. It is completed and sent to the insurer within 28 days of the crash. The Personal Injury Claim Form is for a full insurance claim (eligibility for a full claim is subject to succeeding in proving that the other driver was at-fault in the motor vehicle crash, while this is not the case for the limited claim) [16].

People aged 18 years or older who had sustained injuries in a motor vehicle crash in NSW between March and December 2010 were identified and invited to participate in the study. Participants were excluded if they: a) sustained severe injuries (severe traumatic brain injury or spinal cord injury); b) had an injury requiring hospitalization for more than 7 days; c) had a New Injury Severity Score (NISS) > 8; d) were unable to complete questionnaires by telephone in English; or e) if contact could not be initiated within 60 days of the crash date.

**Fig. 1:**
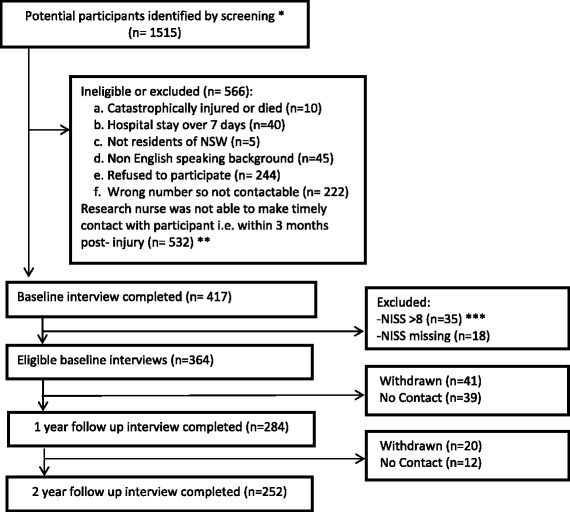
Flowchart of study participation. *These participants were identified as potentially suitable for the study as they were screened and excluded if they: 1) Had claims associated with death and nervous shock; 2) Had severe injuries (defined by the Life Time Care Scheme, NSW i.e. burns, amputation, blindness, spinal cord injury and severe traumatic brain injury); 3) Were aged < 18; 4) Were non-residents of NSW; and 5) Were already 3 months post-injury. ** Due to the limited time the research nurse was employed on this project, there were participants she was not able to contact for participation within a reasonable time period i.e. 3 months post-injury. ***NISS – New Injury Severity Score is determined progressively in the claim process as medical records become available to trained coders at the Motor Accident Authority. Therefore for claims where NISS could not be determined due to insufficient information or score of > 8 by 24 months of injury, were excluded from the analysis. *These participants were identified as potentially suitable for the study as they were screened and excluded if they: 1) Had claims associated with death and nervous shock; 2) Had severe injuries (defined by the Life Time Care Scheme, NSW i.e. burns, amputation, blindness, spinal cord injury and severe traumatic brain injury); 3) Were aged < 18; 4) Were non-residents of NSW; and 5) Were already 3 months post-injury. ** Due to the limited time the research nurse was employed on this project, there were participants she was not able to contact for participation within a reasonable time period i.e. 3 months post-injury. ***NISS – New Injury Severity Score is determined progressively in the claim process as medical records become available to trained coders at the Motor Accident Authority. Therefore for claims where NISS could not be determined due to insufficient information or score of > 8 by 24 months of injury, were excluded from the analysis

A total of 1,515 insurance claims that were lodged between March 2010 and December 2010 were deemed to be potential participants (Fig. 1), and these individuals were invited to participate in the study. An opportunity to opt out of the study within 2 weeks was provided, following which, verbal consent was sought. The study was approved by the University of Sydney Human Research Ethics committee. Of the 1515, 1098 were not eligible or refused to participate. Of the remaining 417 who participated in the baseline interview, 53 were excluded as they had missing NISS or an NISS > 8 (those with severe injury). This left 364 participants that could be included in analyses. Follow-up assessments were completed on 284 (78 % of eligible participants at baseline) at 12 months and 252 (69 % follow-up rate) of 364 enrolled and eligible participants at 24 months (Fig. 1) [16]. For the current report, however, we have data available for all 364 participants surveyed at baseline given that the outcome was time to claim closure, which was obtained by the SIRA as the study progressed.

### Assessment of potential explanatory variables

Study participants were interviewed by telephone on average 56 (range 25–102) days following the date of the accident. Information on socio-demographics, return to work, motor vehicle crash details and pain, disability and health-related quality of life was collected. The interview took nearly 45 min and all interviews were administered by one trained and experienced research nurse. Participants reported on their age, gender, education (tertiary qualified or not tertiary qualified), and pre-injury paid work status was based on being in paid employment (including being self-employed) versus those who were not in paid employment (unemployed, home duties, voluntary work, student or retired).

Trained and experienced coders were used to code the reported injuries. The Abbreviated Injury Scale (AIS) coding system was used to classify the participants into the mild (NISS 1–3) and moderate (NISS 4–8) injury severity groups based on the NISS [17]. NISS data is determined progressively in the claim process as medical records become available to trained coders at SIRA. Data on the type of injury sustained: whiplash (*n* = 224) or fracture (*n* = 30) were collated from the SIRA Personal Injury Registry database. There were, however, 110 participants for which the injury type was not known.

Pre-injury chronic illness was determined by asking participants if they had been diagnosed with any of the following by a doctor: asthma, cancer, heart or circulatory condition, diabetes, mental and behavioral problems, and/or other chronic condition that was not listed. If participants reported that they had had any of the above long term illness for more than 3 months then this was considered as having a chronic illness. Pre-injury chronic pain was characterized by participants reporting that they had been diagnosed with the following for more than 3 months: arthritis, neck and back problems/disorder, and pain.

Participants were asked to describe their general health status prior to the motor vehicle accident, using a five-point Likert scale (excellent, very good, good, fair, or poor). Body mass index was calculated from self-reported height and weight. BMI was classified according to WHO guidelines as ≤ 24.9 kg/m^2^ (normal/underweight) and ≥ 25 kg/m^2^ (overweight/obese). Participants were also asked how many hours they spent in hospital after the crash. They were then dichotomized to those who spent less than 24 h in hospital (non-hospitalized group) and those that spent 24 h or more in hospital (hospitalized group) after the crash.

EQ-5D-3 L was used to measure health related quality of life [18]. The first part of the EQ-5D-3 L has five dimensions: mobility, self-care, usual activities, pain/discomfort and anxiety/depression. Each dimension is divided into three levels of severity: no problem, some problems and major problems. The second part of EQ-5D-3 L is a 20-cm visual analogue scale (VAS), which was modified slightly from the original version with the question (given that it was administered over the phone rather than the participant self-administering EQ VAS): ‘To help you say how good or bad your health state is, I have a scale in front me (rather like a thermometer), on which the best health state you can imagine is marked 100 and the worst health state you can imagine is marked 0’ [18, 19]. The Medical Outcomes Survey Short Form-12 (SF-12) was used as another measure of health-related quality of life [20]. The SF-12 has 12 questions selected from the SF-36 Health Survey [21]. Scoring of the SF-12 provides results on 8 domains (physical functioning, role limitations due to physical problems, bodily pain, general health, vitality, social functioning, role limitations due to emotional problems, and mental health). Two component scores, the physical and mental component summaries (i.e. SF-12 PCS and SF-12 MCS), are derived from the domain scores, the domain scores and component scores are standardized to a mean of 50 and standard deviation of 10. A score below 50 for the SF-12 PCS and MCS is indicative of poor physical and mental health, respectively.

The short form Orebro Musculoskeletal Pain Screening Questionnaire (OMPSQ) was developed as a screening tool for the early identification of persons likely to develop persistent disability from musculoskeletal pain that affects return to work [22]. The Short Form OMPSQ is a 10-item questionnaire, with a possible total score of 100. A score above 50 identifies individuals at high risk of developing poor return to work outcomes [22]. The Pain-Related Self-Statements Scale-Catastrophizing Subscale (PRSS-Catastrophizing) is a nine-item self-report inventory that measures the frequency of a patient’s catastrophic cognitions that may impede the individual’s ability to cope with severe pain [23]. Patients are asked to rate the frequency with which they experience particular catastrophic thoughts during an episode of pain, and the overall score is calculated with a range of 0 (‘almost never’) to 5 (‘almost always’), with higher scores reflecting more frequent endorsement of catastrophic thoughts. The total score for all items is divided by 9 to obtain a mean item score. The PRSS-Catastrophizing is a well validated and widely used measure in clinical chronic pain samples [23, 24].

### Assessment of time to claim closure and other compensation factors

Time to claim closure was determined by subtracting the crash date from the claim settlement date. These dates were derived from the SIRA Personal Injury Register database. Time to claim closure was characterized into three groups: 0–12 months (reference group or early claim settlement); > 12–24 months (medium); and > 24 months (late). We chose to classify claim closure time as 3 distinct groups, based on the insurance regulator (NSW SIRA) advising us that assessing claim closure in this manner is both informative and of practical relevance. Additional analysis also involved assessing time to claim closure as a continuous variable i.e. cumulative months in the compensation system. Other compensation related factors that were collected included whether the participants had lodged a previous claim (yes/no), and whether they engaged a lawyer (yes/no) [9].

### Statistical analysis

Study characteristics of participants at baseline were summarized using descriptive statistics. Univariate multinomial regression analyses was performed in the first instance to identify significant risk factors or covariates (i.e. 2-tailed, *p* < 0.05) for the key study outcome which was time to claim closure: 0–12 months (reference); > 12–24 months; and > 24 months. Potential confounders that were assessed as dichotomized variables included: sex, education level, pre-injury paid work status, lawyer involvement at 12 months, BMI, pre-injury variables (health status, chronic illnesses and pain), hospital admission, NISS scores (mild/moderate), whiplash, and fracture. Age, SF-12 MCS and PCS, EQ VAS, PRSS-catastrophizing, and OMPSQ scores were treated as continuous variables (per 1-unit increase) when assessing whether they were potential confounders of associations with claim closure time. Multivariable multinomial regression analyses selected covariates significantly associated with categories of time to claim settlement, using a forced entry procedure. We report adjusted odds ratio (OR) and 95 % confidence intervals (CIs) for time to claim closure. Additional logistic regression analysis was conducted comparing late claim closure (> 24 months) to medium claim closure (> 12–24 months; reference group). Outcomes were also assessed using Cox proportional survival analysis, an approach similar to that in the study by *Casey* et al. [12] was taken. In this analysis, the measure of outcome is cumulative months from claim notification (inception point) to claim closure. The outcomes of participants still receiving compensation benefits past 24 months post-injury were considered censored. In this analysis, hazard risk ratios (HR) less than 1 indicate an increased risk of time in the compensation system. Conversely a HRR greater than 1 indicates a faster time to claim closure [12]. All statistical analyses were done using SPSS v 21.

## Results

Of the 364 claimants surveyed at baseline, just over half (54 %) had settled their claim within 12 months, while 17 and 30 % took > 12–24 months and > 24 months to settle their claim, respectively. Table 1 shows the study characteristics of participants stratified by claim closure status. Univariate analyses showed that being overweight/obese at baseline, sustaining a moderate/severe injury, and fracture, and lawyer involvement were associated with greater odds of both medium and late claim closure times rather than early claim settlement (< 12 months) (Table 2). OMPSQ and PRSS-catastrophizing scores were positively associated with claim closure times, while SF-12 PCS and EQ VAS scores were all inversely associated with the likelihood of medium or late claim closure versus early claim settlement (Table 2).

**Table 1:** Study characteristics of participants (*n* = 364) stratified by claim closure status

	Time to claim closure		
Parameters^a^	0–12 months	> 12–24 months	> 24 months
	*n* = 195 (54 %)	*n* = 61 (17 %)	*n* = 108 (30 %)
Age	46.0 (17.8)	44.8 (15.0)	44.4 (15.5)
Sex			
Male (*n* = 135)	75 (39 %)	24 (39 %)	36 (33 %)
Female (*n* = 229)	120 (62 %)	37 (61 %)	72 (67 %)
Country of birth			
Australia (*n* = 236)	122 (63 %)	40 (66 %)	74 (69 %)
Other (*n* = 128)	73 (37 %)	21 (34 %)	34 (32 %)
Education			
Not tertiary qualified (*n* = 168)	88 (45 %)	29 (48 %)	51 (48 %)
Tertiary qualified (*n* = 195)	107 (55 %)	32 (53 %)	56 (52 %)
Marital status			
Single (*n* = 150)	79 (41 %)	24 (39 %)	47 (44 %)
Married (*n* = 213)	115 (59 %)	37 (61 %)	61 (57 %)
Pre-injury paid work status			
Unemployed (*n* = 137)	74 (38 %)	25 (41 %)	38 (35 %)
Employed (*n* = 227)	121 (62 %)	36 (59 %)	70 (65 %)
Body mass index			
Underweight/Normal (*n* = 157)	100 (51 %)	17 (28 %)	40 (37 %)
Overweight/obese (*n* = 207)	95 (49 %)	44 (72 %)	68 (63 %)
Smoking			
No (*n* = 213)	116 (60 %)	32 (53 %)	65 (60 %)
Yes (*n* = 149)	78 (40 %)	28 (47 %)	43 (40 %)
Pre-injury health status			
Fair/poor (*n* = 23)	15 (8 %)	3 (5 %)	5 (5 %)
Good/very good/excellent (*n* = 341)	180 (92 %)	58 (95 %)	103 (95 %)
Pre-injury chronic illness			
No (*n* = 218)	121 (63 %)	37 (61 %)	60 (56 %)
Yes (*n* = 146)	74 (38 %)	24 (39 %)	48 (44 %)
Pre-injury chronic pain			
No (*n* = 311)	169 (87 %)	50 (82 %)	92 (85 %)
Yes (*n* = 53)	26 (13 %)	11 (18 %)	16 (15 %)
New injury severity scale			
Mild (*n* = 310)	179 (92 %)	47 (77 %)	84 (78 %)
Moderate (*n* = 54)	16 (8 %)	14 (23 %)	24 (22 %)
Admitted to hospital (≥ 1 night)			
No (*n* = 295)	164 (84 %)	50 (82 %)	81 (75 %)
Yes (*n* = 69)	31 (16 %)	11 (18 %)	27 (25 %)
Whiplash (due to the car crash)			
No (*n* = 139)	84 (43 %)	18 (30 %)	37 (35 %)
Yes (*n* = 224)	111 (57 %)	43 (71 %)	70 (65 %)
Fracture (due to the car crash)			
No (*n* = 333)	188 (96 %)	51 (84 %)	94 (88 %)
Yes (*n* = 30)	7 (4 %)	10 (16 %)	13 (12 %)
OMPSQ score (per 1-unit)	33.3 (23.9)	54.2 (20.1)	55.4 (21.0)
PRSS-catastrophizing score (per 1-unit)	0.7 (1.1)	1.6 (1.3)	1.9 (1.4)
SF-12 PCS (per 1-unit)	41.0 (11.6)	32.2 (8.8)	31.4 (9.2)
SF-12 MCS (per 1-unit)	48.7 (10.4)	46.5 (10.7)	42.8 (12.1)
EQ-5D VAS (per 1-unit)	72.1 (19.8)	60.9 (23.8)	57.0 (21.6)
Lawyer involvement			
No (*n* = 258)	184 (94 %)	33 (54 %)	41 (38 %)
Yes (*n* = 106)	11 (6 %)	28 (46 %)	67 (62 %)
Previous claim			
No (*n* = 252)	138 (71 %)	40 (66 %)	74 (69 %)
Yes (*n* = 111)	56 (29 %)	21 (34 %)	34 (32 %)

Pain-Related Self-Statements Scale-Catastrophizing (PRSS) score; *OMPSQ* short-form orebro musculoskeletal pain screening questionnaire, *PCS* physical component score, *MCS* mental component score, *VAS* visual analogue scale

^a^All parameters are those that are measured at baseline (within 3 months of sustaining the injury), except for lawyer involvement which was assessed throughout the study period

**Table 2:** Univariate multinomial regression analyses showing the association between socio-demographic, psychological, health and injury-related characteristics and time to claim closure (*n* = 364), presented as adjusted odds ratio (OR) and 95 % confidence interval (CI)

Parameters^a^	> 12–24 months to settle claim^b^	> 24 months to settle claim^b^
	OR (95 % CI)	OR (95 % CI)
Age	1.00 (0.98–1.01)	0.99 (0.98–1.01)
Sex		
Male (*n* = 135)	1.0 (reference)	1.0 (reference)
Female (*n* = 229)	0.96 (0.54–1.74)	1.25 (0.76–2.05)
Country of birth		
Australia (*n* = 236)	1.0 (reference)	1.0 (reference)
Other (*n* = 128)	0.88 (0.48–1.60)	0.77 (0.47–1.27)
Education^a^		
Tertiary qualified (*n* = 195)	1.0 (reference)	1.0 (reference)
Not tertiary qualified (*n* = 168)	0.91 (0.51–1.62)	0.90 (0.56–1.45)
Marital status		
Single (*n* = 150)	1.0 (reference)	1.0 (reference)
Married (*n* = 213)	1.06 (0.59–1.91)	0.89 (0.55–1.44)
Pre-injury paid work status		
No (*n* = 137)	1.0 (reference)	1.0 (reference)
Yes (*n* = 227)	0.88 (0.49–1.58)	1.13 (0.69–1.84)
Body mass index		
Underweight/Normal (*n* = 157)	1.0 (reference)	1.0 (reference)
Overweight/obese (*n* = 207)	2.72 (1.46–5.10)	1.79 (1.16–2.90)
Smoking		
No (*n* = 213)	1.0 (reference)	1.0 (reference)
Yes (*n* = 149)	1.30 (0.73–2.33)	0.98 (0.61–1.59)
Pre-injury health status		
Fair/poor (*n* = 23)	1.0 (reference)	1.0 (reference)
Good/very good/excellent (*n* = 341)	1.61 (0.45–5.76)	1.72 (0.61–4.86)
Pre-injury chronic illness		
No (*n* = 218)	1.0 (reference)	1.0 (reference)
Yes (*n* = 146)	1.06 (0.59–1.91)	1.31 (0.81–2.11)
Pre-injury chronic pain		
No (*n* = 311)	1.0 (reference)	1.0 (reference)
Yes (*n* = 53)	1.43 (0.66–3.10)	1.13 (0.58–2.22)
New injury severity scale		
Mild (*n* = 310)	1.0 (reference)	1.0 (reference)
Moderate/severe (*n* = 54)	3.33 (1.52–7.31)	3.20 (1.61–6.33)
Admitted to hospital (≥1 night)		
No (*n* = 295)	1.0 (reference)	1.0 (reference)
Yes (*n* = 69)	1.16 (0.55–2.48)	1.76 (0.99–3.15)
Whiplash (due to the car crash)		
No (*n* = 139)	1.0 (reference)	1.0 (reference)
Yes (*n* = 224)	1.81 (0.97–3.36)	1.43 (0.88–2.33)
Fracture (due to the car crash)		
No (*n* = 333)	1.0 (reference)	1.0 (reference)
Yes (*n* = 30)	5.27 (1.91–14.52)	3.71 (1.43–9.62)
OMPSQ score (per 1-unit)	1.04 (1.03–1.06)	1.04 (1.03–1.06)
PRSS-catastrophizing score (per 1-unit)	1.77 (1.41–2.24)	2.03 (1.66–2.48)
SF-12 PCS (per 1-unit)	0.93 (0.90–0.96)	0.92 (0.90–0.94)
SF-12 MCS (per 1-unit)	0.98 (0.96–1.01)	0.95 (0.93–0.98)
EQ VAS score (per 1-unit)	0.97 (0.96–0.99)	0.97 (0.96–0.98)
Lawyer involvement		
No (*n* = 209)	1.0 (reference)	1.0 (reference)
Yes (*n* = 114)	14.19 (6.44–31.27)	27.34 (13.28–56.26)
Previous claim		
No (*n* = 283)	1.0 (reference)	1.0 (reference)
Yes (*n* = 132)	1.29 (0.70–2.39)	1.13 (0.68–1.89)

Pain-Related Self-Statements Scale-Catastrophizing (PRSS) score; *OMPSQ* short-form orebro musculoskeletal pain screening questionnaire, *PCS* physical component score, *MCS* mental component score, *VAS* visual analogue scale

^a^All parameters are those that are measured at baseline (within 3 months of sustaining the injury), except for lawyer involvement which was throughout the study period

^b^Reference group are those participants who settled their claim in 0–12 months

After multivariable-adjustment, lawyer involvement during the study period, being overweight/obese, presence of whiplash and OMPSQ scores (per 1-unit increase) at baseline were independent predictors of claim settlement at > 12–24 months compared to less than 12 months: 10.4-, 2.9-, 2.4-and 1.0-fold increased odds, respectively (Table 3). Lawyer involvement during the study period was associated with a 21.0-fold greater likelihood of settling a claim at >24 months compared to < 12 months (Table 3). OMPSQ scores were also positively associated with delayed claim closure, OR 1.02 (95 % CI 1.00–1.05). Supplementary logistic regression analysis where the reference group was claim closure at > 12–24 months showed that each per 1-unit increase in SF-12 MCS scores at baseline were associated with reduced odds of delayed claim closure (> 24 months): multivariable-adjusted OR 0.96 (95 % CI 0.92–1.00). Lawyer involvement was also associated with delayed claim closure (> 24 months) compared to settling at > 12–24 months: multivariable-adjusted OR 2.24 (95 % CI 1.11–4.52).

**Table 3:** Multivariable model of factors independently associated with time to claim closure among participants, presented as adjusted odds ratio (AOR) and 95 % confidence intervals (CI)

Predictors^a^	Multivariate-adjusted estimates		
Medium claim closure at >12–24 months^b^	AOR	95 % CI	*P*-value
Age	0.99	(0.97–1.02)	0.544
Sex (ref = Male)	1.11	(0.52–2.34)	0.791
Body mass index (ref = Underweight/Normal)	2.92	(1.36–6.26)	0.006
New injury severity scale (ref = mild)	2.29	(0.83–6.33)	0.110
Admitted to hospital (ref < 1 night)	0.55	(0.20–1.53)	0.525
Whiplash (ref = No)	2.38	(1.06–5.39)	0.037
Fracture (ref = No)	4.25	(1.00–18.16)	0.051
OMPSQ score (per 1-unit)	1.04	(1.01–1.07)	0.006
PRSS-catastrophizing (per 1-unit)	0.97	(0.65–1.45)	0.874
SF-12 PCS (per 1-unit)	0.99	(0.95–1.05)	0.812
SF-12 MCS (per 1-unit)	1.04	(1.00–1.08)	0.077
EQ VAS score (per 1-unit)	1.00	(0.98–1.02)	0.658
Lawyer at 12 months (ref = No)	10.44	(4.29–25.38)	< .001
Late claim closure at > 24 months^b^			
Age	0.99	(0.97–1.01)	0.266
Sex (ref = Male)	1.44	(0.71–2.91)	0.318
Body mass index (ref = Underweight/Normal)	1.60	(0.82–3.14)	0.169
New injury severity scale (ref = mild)	2.29	(0.90–5.86)	0.084
Admitted to hospital (ref < 1 night)	1.03	(0.43–2.46)	0.948
Whiplash (ref = No)	1.60	(0.78–3.31)	0.202
Fracture (ref = No)	1.87	(0.48–7.32)	0.369
OMPSQ score (per 1-unit)	1.02	(1.00–1.05)	0.069
PRSS-catastrophizing (per 1-unit)	1.01	(0.70–1.46)	0.947
SF-12 PCS (per 1-unit)	0.98	(0.94–1.03)	0.481
SF-12 MCS (per 1-unit)	1.00	(0.97–1.04)	0.958
EQ VAS score (per 1-unit)	0.99	(0.97–1.01)	0.271
Lawyer at 12 months (ref = No)	20.97	(9.40–46.79)	< .001

Pain-Related Self-Statements Scale-Catastrophizing (PRSS) score; *OMPSQ* short-form orebro musculoskeletal pain screening questionnaire, *PCS* physical component score, *MCS* mental component score, *VAS* visual analogue scale

^a^All parameters are those that are measured at baseline (within 3 months of sustaining the injury), except for lawyer involvement which was throughout the study period

^b^Reference group are those participants who settled their claim in 0–12 months

Additional Cox proportional survival analysis was performed to assess the predictors of time to claim closure (cumulative months in the compensation system). Table 4 shows that compared to claimants who were normal/underweight those who were overweight or obese had an increased risk of prolonged exposure to the compensation system, multivariable-adjusted HRR 0.77 (95 % CI 0.59–1.00). Similarly, lawyer involvement was also independently associated with risk of slower time to claim closure, multivariable-adjusted HRR 0.28 (95 % CI 0.19–0.40). Finally, each 1-unit increase in baseline SF-12 PCS scores was associated with faster time to claim closure, HRR 1.02 (95 % CI 1.00–1.04), while OMPSQ scores (per 1-unit increase) at baseline were inversely associated with time to claim closure, multivariable-adjusted HRR 0.99 (95 % CI 0.98–1.00).

**Table 4:** Multivariable Cox regression model of factors associated with time to claim closure (cumulative months)

Predictors	Adjusted HRR (95 % CI)	*P*-value
Age	1.01 (1.00–1.01)	0.214
Sex (ref = Male)	0.85 (0.64–1.12)	0.248
Body mass index (ref = Underweight/Normal)	0.76 (0.58–0.99)	0.044
New injury severity scale (ref = mild)	0.78 (0.52–1.19)	0.257
Admitted to hospital (ref < 1 night)	1.14 (0.79–1.65)	0.480
Whiplash (ref = No)	0.82 (0.62–1.09)	0.172
Fracture (ref = No)	0.77 (0.43–1.36)	0.363
OMPSQ score (per 1-unit)	0.99 (0.98–1.00)	0.022
PRSS-catastrophizing (per 1-unit)	1.00 (0.85–1.18)	0.986
SF-12 PCS (per 1-unit)	1.02 (1.00–1.04)	0.044
SF-12 MCS (per 1-unit)	1.01 (0.99–1.02)	0.467
EQ-5D VAS score (per 1-unit)	1.00 (0.99–1.01)	0.773
Lawyer at 12 months (ref = No)	0.28 (0.19–0.40)	< 0.001

*HRR* Hazard Risk Ratio

## Discussion

Our cohort study showed that 30 % of participants who had sustained minor road traffic injuries took > 24 months to settle their claim. Health-related factors such as being overweight/obese, baseline presence of whiplash, and higher OMPSQ scores were all independent predictors of delayed claim closure. Consulting a lawyer during the study period was also a strong prognostic indicator of protracted time in the compensation system (i.e. > 24 months).

Around a third of claimants in our study still had not settled by 24 months. This rate is lower than the previously reported 50 % of Australian adults with whiplash who had delayed claim settlement (> 24 months) [25, 26]. However, it is similar to another Australian study, where 25 % of participants with whiplash remained in the compensation system for longer than 24 months [12], and the 30 % observed in a New Zealand study of back pain claimants who received compensation past 12 months. Given that the ‘time taken to deal with a claim’ is associated with stresses that could hinder recovery [6, 12] and negatively impact health status in the longer term [4], our finding that ~30 % of claimants remained within the compensation system past 24 months is noteworthy.

Our finding that being overweight or obese at baseline was associated with delayed claim settlement (>12–24 months), contrasts with data from a Canadian study where being overweight or obese at baseline did not independently influence time to claim closure [27]. Nevertheless, our findings are in agreement with other studies which show that excess weight is a likely risk factor for chronic pain and disability [28, 29], and that weight reduction is a potentially valuable treatment option for e.g. the rehabilitation of spinal pain [27, 30]. Moreover, obesity in claimants could lead to delayed claim closure through several mediating pathways involving numerous physical and psychological comorbidities [31, 32] and/or a poorer general health status [27, 33]. However, in our study the relationship between overweight/obesity and delayed claim closure persisted even after adjusting for baseline physical and mental health measures (e.g. SF-12 PCS and MCS), and pre-injury health factors (i.e. presence of chronic illness and/or chronic pain), hence, suggesting other yet unmeasured or unaccounted factors could be underlying this association in our study.

We observed that whiplash sustained in the crash was associated with greater odds of settling the claim at >12–24 months, and this association was independent of injury severity. For claimants with whiplash injury in particular, due to the lack of clinical findings, claimants could encounter conflicting medical opinions, unsuccessful therapies, and stigma/distrust in the process of documenting their suffering and disability under a fault-based compensation scheme [34]. Hence, this potentially adversarial environment could delay claim settlement. Nevertheless, whiplash was not an independent predictor of claim closure > 24 months. This finding suggests that the nature of the injury and the associated pain and lowered physical functioning could play a role in the medium-term, because of claimants needing to access medical care (hospitalization and rehabilitation programs), which could directly influence claim settlement time. However, in the longer-term, factors other than the type of injury, such as psychosocial factors might be more important predictors of prolonged exposure to the compensation system [25].

The OMPSQ has been promoted as a tool to screen for psychosocial risk factors associated with delayed recovery [22], and we previously showed it to be associated with persistent pain [35] and delayed return to work [36] following an injury. Hence, the finding that OMPSQ scores was a strong and independent predictor of delayed claim closure (> 24 months) was expected. Specifically, this particular tool screens for risk factors such as emotional state, fear-avoidance beliefs and coping strategies [22], and these risk factors could make claimants more vulnerable to the compensation system generated stressors (e.g. numerous assessments) which in turn could adversely affect their ability to deal with the claim and exit the scheme faster [12].

It was not surprising that lawyer involvement was a strong and independent predictor of claimants receiving compensation benefits past 24 months, given that other studies have also observed this link [12, 25]. Moreover, our findings are also in agreement with research showing that legal involvement in the compensation process has been associated with several negative outcomes and delayed recovery [12, 37, 38]. The potential reasons underlying this association are not clear. It has been speculated that lawyers could be choosing cases based on economic viability, that is, cases having a permanent injury and higher impairment levels represent more economically viable clients [39]. It has also been suggested that some advocates encourage claimants to remain inactive in order to maximize compensation [1, 39]. Another reason for the association might due to the effects of prolonged exposure to the compensation system, particularly the enhanced adversarial nature of this system when lawyers become engaged in a claim [11, 12]. For instance, claimants report that they require lawyers to assist them with steering through the ‘complexities of the insurance arrangements’ and to help them negotiate with insurers [11, 12]. However, it is also possible that the observed relationship between lawyer involvement and delayed claim closure could be mediated by the above two pathways occurring in tandem. Hence, creating a vicious cycle where claimants find themselves tied to the compensation system [12].

Our study has several strengths, including its longitudinal cohort design and sample size. We also captured a wide spectrum of explanatory variables such as demographic indices, psychosocial measures, and injury- and compensation-related factors. Nevertheless, our study is not without its limitations. First, when analyzing certain associations (e.g. with lawyer involvement), the confidence intervals were very wide, hence, we highlight that these findings could be due to chance and require further confirmation (although the association between lawyer involvement and claim closure was consistent with previous literature). Moreover, we had a very small number of participants who had 24-month follow-up data (*n* = 252), and we are likely to have had insufficient study power to detect certain associations and thus, our findings need to be interpreted with caution. Second, we cannot discount the influence of residual confounding, particularly, as we did not collect information on system generated stressors (e.g. frequency and type of dispute, frequency of medico-legal assessments) [12], which could have influenced observed associations. Third, our findings are not likely to be generalizable to all populations, as personal injury schemes are heterogeneous, hence, the extent to which our results are applicable to specific jurisdictions (e.g. no-fault scheme) should be carefully considered [12]. Finally, the use of time-to-claim closure as an outcome has been criticized, as its association with recovery following an injury has not been established [15, 40]. However, in the current study we are not implying that claim closure means recovery, we wanted to assess the independent predictors of delayed claim settlement, which for example, is of more practical use to insurers and claim coordinators when developing interventions [12].

## Conclusions

In summary, this cohort study reinforced the notion that claim settlement is a complex and multifactorial process. A claimant profile of unhealthy weight status, presence of whiplash at baseline, higher OMPSQ scores and legal involvement appear to independently influence time-to-claim closure. These data strengthen the case for the routine collection of other health measures by insurers at the time of claim lodgment in order to increase their knowledge on both longer-term health and compensation-risk profiles of their claimants [12]. More importantly, these study findings suggest that a wide spectrum of prognostic factors may need to be considered by compensation insurers in order to implement interventions aimed at minimizing the impact of these factors, and hence, leading to potential reductions in claim duration and claim costs.

## Abbreviations

AIS: abbreviated injury score
BMI: body mass index
MCS: mental component summary
OMPSQ: Orebro musculoskeletal pain screening questionnaire
PCS: physical component summary
PRSS: pain-related self-statements scale
SIRA: state insurance regulatory authority
VAS: visual analogue scale
